# Atrazine Binds to the Growth Hormone–Releasing Hormone Receptor and Affects Growth Hormone Gene Expression

**DOI:** 10.1289/ehp.0900738

**Published:** 2010-06-08

**Authors:** Walid D. Fakhouri, Joseph L. Nuñez, Frances Trail

**Affiliations:** 1 Department of Plant Biology; 2 Department of Plant Pathology and; 3 Neuroscience Program, Michigan State University, East Lansing, Michigan, USA

**Keywords:** atrazine, dexamethasone, GHRHR, GHRH receptor, growth hormone, luteinizing hormone, postnatal pituitary cells, prolactin, rat

## Abstract

**Background:**

Atrazine (ATR), a commonly used herbicide in the United States, is widely distributed in water and soil because of its mobility through ecosystems and its persistence in the environment. ATR has been associated with defects in sexual development in animals, but studies on mammalian systems have failed to clearly identify a cellular target.

**Objectives:**

Our goal in this study was to identify a ligand-binding receptor for ATR in pituitary cells that may explain the mechanism of action at the gene expression level.

**Methods:**

We used pituitary cells from postnatal day 7 male rats and pituitary cell lines to study the effect of ATR on gene expression of growth hormone (*GH*), luteinizing hormone (*LH*), and prolactin (*PRL*) at RNA and protein levels. ^14^C-ATR was used to determine its specific binding to the growth hormone–releasing hormone receptor (GHRHR). The effect of ATR on structural proteins was visualized using immunofluorescent *in situ* staining.

**Results:**

The treatment of rat pituitary cells with ATR, at environmentally relevant concentrations (1 ppb and 1 ppm), resulted in a reduction of GH expression. This effect appeared to result from the inhibition of *GH* gene transcription due to ATR binding to the GHRHR of the pituitary cells.

**Conclusions:**

Identification of GHRHR as the target of ATR is consistent with the myriad effects previously reported for ATR in mammalian systems. These findings may lead to a better understanding of the hazards of environmental ATR contamination and inform efforts to develop guidelines for establishing safe levels in water systems.

Atrazine (ATR) is the most commonly used herbicide in the United States, where it has been applied to the control of broad-leaf weeds in a wide range of crops since the 1960s (Mills 1998; U.S. Environmental Protection Agency 2003). In the European Community, ATR use was banned in 2004 (European Commission 2004). The widespread application of ATR to agricultural systems has had a major impact on water and soil quality because of its mobility through ecosystems and its persistence in the environment (Hayes 1993; Hayes et al. 2002b, 2006; Koskinen and Clay 1997). The effect of ATR on animals has been difficult to elucidate. Some studies of whole-animal exposure have correlated low environmental concentrations (0.1–1.0 ppb) with alterations in sexual development in *Xenopus laevis* (Hayes et al. 2002a, 2002b, 2006; Withgott 2002), olfactory-mediated behaviors in goldfish (Sagolio and Trijasse 1998), and mammary tumors in rats (Wetzel et al. 1994). However, other studies have found that environmentally relevant doses of ATR had little effect on development in *Xenopus laevis* and other animals, whereas exposure to higher doses resulted in gonadal abnormalities (Carr et al. 2003; Gammon et al. 2005).

The debate over the toxicological importance of ATR has stimulated research to identify possible mechanisms of endocrine interference. Previous studies have focused on steroid hormone receptors and nuclear receptors as targets for ATR and have eliminated the following receptors as targets: estrogen, neuronal GABA, peroxisome proliferator activator, retinoid-related orphan, androgen, and glucocorticoid (Connor et al. 1996; Devos et al. 2003; Hooghe et al. 2000; Shafer et al. 1999; Tennant et al. 1994). ATR has been shown to inhibit the secretion of gonadotropin- releasing hormone from the hypothalamus in female rats, which leads to a reduction in luteinizing hormone (LH) released from the pituitary gland (Ashby et al. 2002). ATR has been reported to impair LH release in female rats without altering hypothalamic estrogen receptor function (McMullin et al. 2004), whereas inhibition of LH in male rats has been linked to reduction of testosterone (Trentacoste et al. 2001). Studies have suggested that toxic effects of ATR on the nervous system and on the induction of mammary tumors are linked to altered expression of prolactin (PRL) (Missale et al. 1996; O’Connor et al. 2000; Sagrillo and Elmanoff 1998). However, variable effects of ATR on the hypothalamic control of pituitary–ovarian functions and specifically on PRL have been reported (Cooper et al. 2000; O’Connor et al. 2000; Stoker et al. 1999), suggesting that ATR modulation of PRL may be age and sex dependent.

Effects of ATR on fruiting body development of the filamentous fungus *Sclerotinia sclerotiorum* were reported by Casale and Hart (1984). We initiated research to determine the basis of these effects using gene expression profiles. During those studies, we identified a transcript [GenBank accession no. GO066061 (National Center for Biotechnology Information 2010)] that had increased levels in treated tissue and showed slight homology to the growth hormone–releasing hormone receptor (*GHRHR*) gene (Fakhouri WD, Trail F, unpublished data). Studies of this gene are in progress. However, intrigued by those findings, we chose to examine the binding specificity of ATR to the GHRHR and the effects on the expression of its target gene, growth hormone (*GH*), and the expression of other pituitary hormonal balance genes.

## Materials and Methods

### Pituitary cell culture and treatments

All cell culture media components and solutions were obtained from Invitrogen (Carlsbad, CA) unless otherwise stated. All other chemicals were obtained from Sigma Chemical Co. (St. Louis, MO), except RU38486 (a generous gift from S.M. Breedlove, Michigan State University). We obtained postnatal day (PND) 7 male rats from breeder females (Sprague-Dawley; Charles River Labs, Wilmington, MA). Animal use procedures were approved by the Michigan State University All-University Committee on Animal Use and Care and followed National Institutes of Health guidelines (Institute of Laboratory Animal Resources 1996). Animals used in this study were treated humanely and with regard for alleviation of suffering.

Pituitary glands from PND7 male rats were dissected into HBSS+ [88 mL sterile H_2_O, 10 mL Hank’s balanced salt solution (Ca^2+^ and Mg^2+^ free) 10×, 1 mL HEPES buffer (1.0 M, pH 7.3), 1 mL antibiotic/antimycotic 100× liquid (Sigma-Aldrich, St. Louis, MO)], and additional HBSS+ was added to bring the total volume to 4.5 mL. Trypsin was added to 0.28% (wt/vol), and tissue was incubated at 37°C for 15 min. The supernatant was discarded and the tissue washed with HBSS+. The digestion procedure was repeated a second time. Cells were dissociated by trituration, with cell number and viability determined by trypan blue exclusion. Cells were distributed to plates with 25-mm poly-l-lysine–coated coverslips at a density of 300,000 cells per coverslip. Coverslips were then placed in 60-mm dishes containing 4 mL plating medium [86 mL minimal essential medium,10 mL horse serum, 3 mL glucose (filter sterilized, 20%), 1 mL sodium pyruvate (100 mM)], and cells were allowed to adhere to the coverslips for 4 hr in 5% CO_2_ at 37°C. The coverslips were then moved into 60-mm dishes filled with Neurobasal+ [1 mL B-27 supplement, 1 mL antibiotic/antimycotic 100×, 125 μL l-glutamine, and filled to 50 mL with Neurobasal A (phenol red free)]. For competitive binding assays, cells were not distributed to plates but were maintained in HBSS+ until used.

Cultured cells were treated with ATR, dexamethasone (Dex), RU38486, and/or rat growth hormone–releasing factor [GHRF (a 43mer peptide); molecular weight, 5,232 Da; > 95% HPLC purity] to determine the competitive inhibition of each compound on gene expression. All treatments were initiated on day 0 *in vitro.* Cultures were treated with one of eight individual/combinations of agents: DMSO (vehicle); ATR [1 ppm (4.6 μM) or 1 ppb (4.6 nM)]; Dex (10 μM); RU38486 (10 μM); Dex (10 μM) + ATR (1 ppm); RU38486 (10 μM) + ATR (1 ppm); GHRF [0.1 ppm (20 nM), 0.5 ppm (0.1 μM), 1.0 ppm (0.19 μM), 2.0 ppm (0.38 μM), or 4.0 ppm (0.76 μM); or GHRF (0.1, 0.5, 1.0, 2.0, or 4.0 ppm) + ATR (1 ppm). For the combination treatments, the first agent was administered 30 min before the the second agent. All chemicals were dissolved in DMSO such that the final DMSO concentration in the treatment was < 0.5% (vol/vol).

For real-time quantitative polymerase chain reaction (qPCR) and competitive inhibition assays, cells were harvested and rinsed with 0.1 M phosphate-buffered saline (PBS), and subsequently covered with 75 μL RIPA (radioimmunoprecipitation assay) buffer. Cells were then removed from the coverslip, along with the liquid, and stored at −80°C. When cells were harvested for ATR binding-affinity assays, only 1 mL HBSS+ was added to the dissociated cells. The trypan blue exclusion method was used to determine cell number in the 1-mL volume of HBSS+, and cells were counted with a hemocytometer.

### Real-time qPCR analysis of gene expression

We extracted total RNA from treated and untreated pituitary cells. Frozen or fresh cells were dipped briefly in liquid nitrogen and macerated with a pestle in 500 μL TriZol reagent (Invitrogen) using fine glass beads (diameter, 0.1 mm). The extraction and purification steps and subsequent DNase (Roche Applied Science, Indianapolis, IN) treatment were performed according to the manufacturer’s directions. To screen for the presence of DNA contamination, we performed standard PCR using primers specific for each gene. The amount and purity of the total RNA were determined by spectrophotometer readings at 260–280 nm, and equivalent amounts of RNA from each treatment were used for the reverse transcriptase reaction using the Superscript III enzyme (Invitrogen). The real-time qPCR–specific primers for each gene were designed according to the consensus cDNA sequence from GenBank (National Center for Biotechnology Information 2010) using Primer Express Software (ABI700 Prism Software, ABI, Foster City, CA). We used the following primers: for *GH* gene, forward (F), 5′-CAAAGAGTTCGAGCGTGCCTA, reverse (R), 5′TGGGATGGTCTCTGAGAAGCA; *LH* gene F, 5′-CTGTGTGGAGCGGGATTCA, R, 5′-TGCAGGTGGACGACATCAAG; *PRL* gene, F, 5′-ACCATGCTATGTCACGGCTC, R, 5′-CAGGTGCTGGAGTTCCTCGA; tubulin gene, F, 5′-TACCCAGACCGCATCATGAA, R, 5′-GAAAGGGTGGCATTATAGGGC; actin gene, F, 5′-ACGGTCAGGTCATCACTATCGG, R, 5′-TGCCACAGGATTCCATACCC; and histone H3 gene, F, 5′-GGTAAAGCACCCAGGAAACA, R, 5′-ACCAGGCCTGTAACGATGAG. Real-time qPCR normalization and analysis were performed according to Huggett et al. (2005) and Vandesompele et al. (2002).

For each experiment, results from the treatment replicates were averaged, and then the amount of mRNA of each gene was normalized to the mRNA of the histone H3 gene. We calculated the SE of each treatment based on the variation among the replicates of each tested gene, normalized to the SE of histone H3 in those samples. Each biological experiment was repeated at least twice.

### Protein levels in ATR-treated pituitary cell cultures

We determined protein levels of GH, LH, PRL, and the GHRHR in the AtT-20 mouse pituitary tumor cell line [American Type Culture Collection (ATCC), Manassas, VA]. Cell cultures were treated with one of four agents—DMSO (vehicle), Dex (10 μM), ATR [1 ppm (4.6 μM)], or ATR [1 ppb (4.6 nM)]—with three replicates for each treatment. In another approach to block adrenocorticotropic hormone (ACTH) and pituitary adenylate cyclase–activating protein (PACAP) receptors, we quantified protein levels of GH in the rat GH3 pituitary tumor cell line (ATCC) after treatment with 5 or 10 μL ACTH receptor antibody (Santa Cruz Biotechnology, Santa Cruz, CA) at a concentration of 0.2 mg/mL, 15 min before treatment with 1 ppm ATR. Similarly, the GH3 pituitary cell line was treated with 5 or 10 μL PACAP receptor antibody (Santa Cruz Biotechnology) at a concentration of 0.2 mg/mL and incubated at 37°C for 15 min, followed by treatment with 1 ppm ATR. In both pituitary cell lines, protein levels of GH were assayed 2–4 hr after treatment with ATR at 1 ppm and 1 ppb. Rabbit anti-histone antibody was used as a negative control.

After treatment, cell cultures were transferred to Eppendorf tubes and harvested by centrifugation at 2,000 rpm for 5 min. Cells were washed twice with cold PBS and then lysed in 400 μL RIPA buffer, 5 μL protease inhibitor, and 5 μL EDTA for 5 min. Lysed cells were sonicated for 1 min on ice, centrifuged for 10 min at 4°C and the supernatant was transferred into new vials. Equivalent amounts of total protein from each sample were used for SDS-PAGE Western blot analysis. The housekeeping genes β-actin and β-tubulin were used as loading controls. Rabbit anti-rat GH, anti-rat LH, and anti-mouse PRL antibodies were obtained from the National Institute of Diabetes and Digestive and Kidney Diseases National Hormone and Peptide Program. Rabbit anti-human GHRHR antibodies were purchased from Abcam (Cambridge, MA). Goat anti-rabbit antibodies conjugated to horseradish peroxidase (Bio-Rad, Hercules, CA) were used in our chemiluminescence assay. This experiment was repeated twice.

### Competitive binding of ^14^C-ATR and GHRF (43mers) to pituitary cells

The binding affinity of ATR-ring-UL-^14^C (molecular weight, 215.7 Da; ≥ 95% HPLC purity, 25 mCi/mmol; Sigma Chemical Co.) to pituitary cells was assayed after 0.5, 1.0, 2.0, and 3.0 hr at 37°C. For each time point, three tubes containing pituitary cells (1.5 × 10^7^ cells/mL), in 500 μL HBSS, were treated with 1.0 ppm ^14^C-ATR. After incubation for the indicated time, cultures were poured over a fiberglass filter disk (2 cm diameter) in a Buchner funnel and washed with 2 mL TCA buffer (trichloroacetic acid 10% plus sodium pyrophosphate 1%) under vacuum. The filter disks were transferred into separate glass vials containing 5 mL scintillation cocktail (Econo-Safe, Research Products International Corp., Mount Prospect, IL). The vials were shaken briefly and loaded into counting racks. The radioactivity of each vial was measured using a Beckman Coulter LS6500 scintillation counter (Beckman Coulter, Brea, CA). Pituitary cells not exposed to ^14^C-ATR were included as negative controls. To determine how long ^14^C-ATR takes to reach saturation binding to the pituitary cells, we monitored the binding of 1.0 ppm ^14^C-ATR to pituitary cells over a 3-hr time course. No significant increase in ^14^C-ATR binding, as measured by radioactivity associated with cells, was observed after a 0.5-hr incubation.

To test the ability of GHRF to competitively inhibit the binding of ^14^C-ATR, we simultaneously added both GHRF and ^14^C-ATR to pituitary cells (in a total volume of 20 μL for both compounds per 500 μL cell culture). Competition assays were performed using 1.0 ppm ^14^C-ATR and increasing concentrations of GHRF (0.1–4.0 ppm). The cells were incubated at 37°C for 0.5 hr and then processed as described above. As a positive control, we included a competitive inhibition assay with unlabeled ATR and ^14^C-ATR. To determine the linear correlation between GHRF and ATR concentrations to the bound/free ^14^C-ATR ratio, the results were analyzed using linear curve-fitting analysis.

### Immunofluorescent labeling of actin and tubulin

After treatment, culture medium was replaced with warm fixative (4% paraformaldehyde with 5% sucrose in 0.1 M PBS) for 10 min, followed by a rinse in 0.1 M PBS and 1 hr in 50% ethanol at 4°C. Cells were rinsed; then blocking solution (10% normal goat serum, 0.1% Triton in 0.1 M PBS) was added at room temperature (22–24°C) for 0.5 hr. One of the following primary antibodies was used: mouse monoclonal anti-actin antibody (1:1,000) or mouse monoclonal anti-tubulin (β-III isoform) antibody (1:1,000; both antibodies from Chemicon, Temecula, CA). Antibodies were diluted in 10% normal goat serum in 0.1 M PBS; each culture was incubated with the primary antibody for 2 hr at room temperature. Tissue was rinsed and then exposed to the secondary antibody (fluorescein-conjugated goat anti-mouse IgG, 1:2,500; Vector, Burlingame, CA); tissue was rinsed, and Vectastain Elite ABC reagents (Vector) were added according to the manufacturer’s directions. The cells on the coverslips were rinsed, dehydrated, and mounted on a coverslip using the aqueous PVA-DABCO antifading mounting medium (Sigma-Aldrich). Cells were visualized using an inverted Nikon TE2000-U microscope (Nikon, Melville, NY) with a Photometrics Cascade 512B camera (Photometrics, Tucson, AZ) and the Metamorph imaging system (version 6.2; Universal Imaging, Downingtown, PA).

## Results

### Quantification of gene expression

GH, LH, and PRL are major hormones of the anterior pituitary gland that are regulated by specific releasing hormones that bind to the corresponding releasing hormone receptor in the pituitary gland (Childs et al. 1994a, 1999). We examined the effect of ATR on the expression of *GH*, *LH*, and *PRL* genes in cultured rat pituitary cells by treating cells with ATR at environmentally relevant levels (low, 1.0 ppb; high, 1.0 ppm) and monitoring transcript levels to detect shifts in expression.

**Figure 1:**
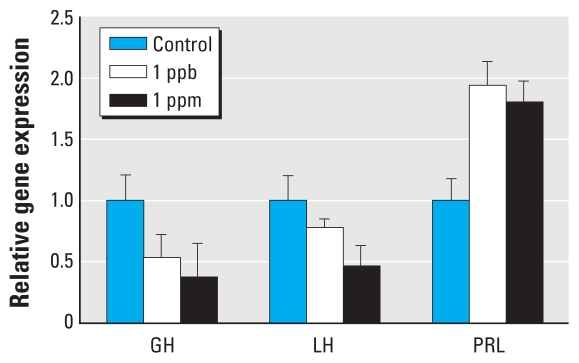
Expression (mean ± SE) of *GH*, *LH*, and *PRL* genes in rat pituitary cells treated with DMSO (control) or with 1.0 ppb or 1.0 ppm ATR. Cells were harvested 72 hr after treatment, and gene expression was measured by real-time qPCR; data were normalized to levels of histone H3 mRNA.

Previous work has demonstrated that neonatal ATR exposure affects the sexual development of frogs, turning them into hermaphrodites (having sexual characteristics of both males and females) (Hayes et al. 2002b; Kniewald et al. 2000). ATR may likewise affect the neuroendocrine axis in mammals. The critical period for sexual differentiation of the rodent brain occurs between embryonic day 18 and PND10 (Arnold and Breedlove 1985). Given that ATR exposure may have the potential to affect sexual differentiation of the rodent brain and that preliminary investigations suggest that the survival of pituitary cells in culture is maximal at the end of the first postnatal week (Nuñez JL, personal communication), we cultured pituitary cells on PND7. Exposure of pituitary cells to 1.0 ppm ATR resulted in a remarkable reduction in the mRNA levels of genes encoding *GH* and *LH* (Figure 1). However, levels of *PRL* mRNA increased in the treated cells compared with controls.

**Figure 2:**
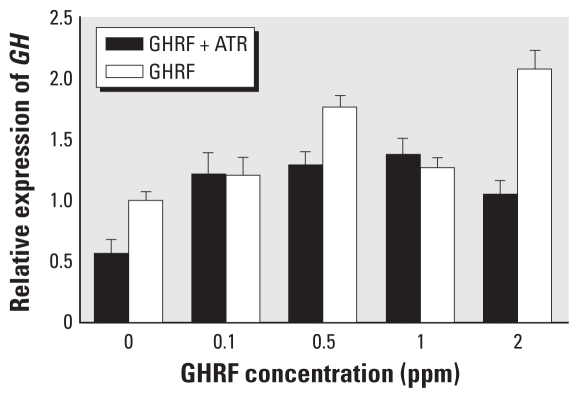
Expression (mean ± SE) of the *GH* gene in pituitary cells 72 hr after treatment with GHRF alone, ATR (1 ppm) alone, or ATR combined with increasing concentrations of GHRF (0.1, 0.5, 1.0, or 2.0 ppm). Treatments with vehicle (DMSO) or ATR alone were used as controls. Data were normalized to levels of histone H3 mRNA for each treatment.

To determine whether ATR effects are mediated through the GHRHR, we used rat GHRF, a 43-amino acid peptide that binds specifically to GHRHR (Bloch et al. 1983; Montero et al. 2000; Spiess et al. 1983; Thorner et al. 1983). Previous studies have shown that GHRF binds to GHRHR and up-regulates the expression of the *GH* gene in healthy human and rat pituitary cells (Bloch et al. 1983; Montero et al. 2000; Thorner et al. 1983) with an optimal range of 1–3 μg/kg body weight *in vivo* and 0.1–10 nM *in vitro* (Thorner et al. 1983; Velicelebi et al. 1986). Serum levels of PRL and LH were not increased after administration of human GHRF at 1 μg/kg body weight (Thorner et al. 1983). We treated pituitary cells with 1.0 ppm ATR alone and combined with increasing levels of GHRF. *GH* expression was reduced in ATR-treated cells compared with controls, but *GH* expression did not appear to be inhibited in cells treated with ATR and GHRF (Figure 2), suggesting that GHRF may displace ATR binding to the GHRHR. Contrary to expectations, *GH* expression was lower after treatment with 1 ppm GHRF compared with 0.5 ppm GHRF. Furthermore, *GH* expression was also slightly reduced in cells treated with ATR and 2 ppm GHRF compared with cells treated with ATR and 1 ppm GHRF. One possible explanation is variation in responses among different batches of primary pituitary cells; however, *GH* expression was two times higher in the cells treated with ATR and 2 ppm GHRF compared with ATR alone. Homeostatic amounts of GH or GHRH may vary among culture runs. This variation could be avoided by using a pituitary cell line. However, cultures of cell lines may also result in changes that do not reflect innate tissue responses.

### Protein levels of GH, LH, PRL, and GHRHR in ATR-treated pituitary cell culture

**Figure 3:**
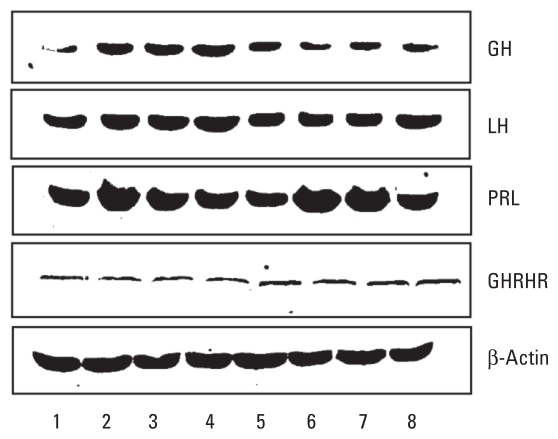
Protein levels of GH, LH, PRL, and GHRHR assayed in mouse AtT-20 pituitary cell culture 30 min after treatment with vehicle (DMSO; lanes 1 and 2), Dex (10 μM; lanes 3 and 4), ATR (1 ppm; lanes 5 and 6), or ATR (1 ppb; lanes 7 and 8). β-Actin protein was used as an internal loading control.

To test the effect of ATR on the final product of *GH*, *LH*, *PRL*, and *GHRHR* genes, we used Western blot analysis to detect the amount of proteins in pituitary cells 30 min after treatment. The amount of GH and LH protein in AtT-20 pituitary cell cultures was slightly reduced in cells treated with 1.0 ppm and 1.0 ppb ATR compared with the negative control (treated with the vehicle alone). In contrast, the amount of PRL was slightly increased in ATR-treated cells. The protein level of GHRHR was similar in all treatment groups (Figure 3).

### Competitive binding of ^14^C-ATR and GHRF (43mers) to pituitary cells

**Figure 4:**
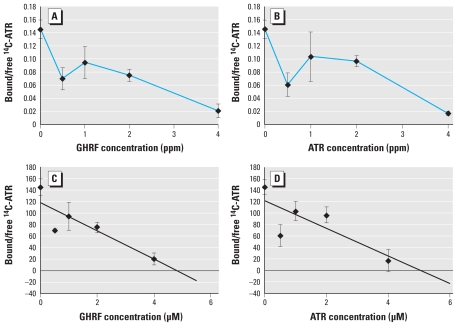
Competitive binding of GHRF to pituitary cells after initial treatment with ^14^C-ATR for 30 min. The labeled pituitary cells were treated with increasing concentrations of GHRF (*A*) or unlabeled ATR (*B*). The correlation between GHRF (*C*) and ATR (*D*) concentrations and the bound/free ^14^C-ATR ratio was analyzed using the linear curve-fitting method. Data are mean ± SE.

Previous studies on GHRHR binding have evaluated displacement of radiolabeled ligands at concentrations ranging from 30 to 80 μg protein/mL (or 0.2 nM) by non-radiolabeled substances at concentrations ranging from 10^−6^ to 10^−12^ M (Rekasi et al. 2000; Varga et al. 1999). To determine whether GHRF displaces ATR on the cell surface, we performed competitive binding studies using ^14^C-radiolabeled ATR. Binding of ^14^C-ATR to pituitary cells reached saturation 0.5 hr after incubation [see Supplemental Material, Figure 1 (doi:10.1289/ehp.0900738)]. If ATR binds to the GHRHR, GHRF should displace ATR on the receptor. Therefore, we performed a competitive binding assay between ^14^C-ATR and GHRF (Figure 4). Pituitary cells treated with ^14^C-ATR and increasing concentrations of GHRF had reduced radioactivity compared with cells treated with ^14^C-ATR alone, which suggests that ^14^C-ATR and GHRF were competing for the same receptor (Figure 4A,C). Unlabeled ATR (0.5–4.0 ppm) also displaced ^14^C-ATR in a concentration-dependent manner (Figure 4B,D).

### Competitive effect of Dex and RU38486 on GH expression

**Figure 5:**
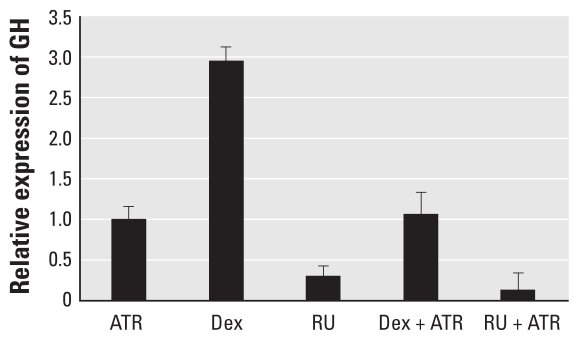
Expression of the *GH* gene in pituitary cells 72 hr after treatment with ATR (1 ppm), Dex (10 μM), RU38486 (RU; 10 μM), Dex + ATR or RU + ATR. Data were normalized to histone H3 mRNA; data are mean + SE.

To further test the role of ATR in affecting *GH* expression through GHRHR, we treated cells with ATR, Dex, or RU38486, alone or combined because Dex and RU38486 act as an agonist and an antagonist, respectively, to *GH* expression. Dex stimulates the expression of the *GH* gene by increasing expression of the *GHRHR* gene in pituitary cells through down-regulation of somatostatin receptor–mediated inhibition of the GHRH protein (Tamaki et al. 1996; Xu et al. 1995). RU38486 reduces the expression of the *GH* gene by repressing the expression of the *GHRHR* gene as a consequence of glucocorticoid receptor repression (Ohyama et al. 1998). Dex alone considerably induced *GH* expression in pituitary cells, but *GH* expression in response to Dex and 1.0 ppm ATR was comparable to expression in response to ATR alone (Figure 5). RU38486, which normally antagonizes the expression of the *GH* gene, caused enhanced repression when combined with ATR; however, the repression does not seem to be remarkably different compared with RU38486 alone because of variation within the technical replicates (Figure 5). The results of these experiments may suggest that RU38486 and ATR act in concert to lower the expression of *GH* and that ATR can mask Dex effects. If ATR acts through the GHRHR, then ATR would enhance the effects of RU38486 and suppress the effects of Dex, which is what we observed.

### ATR effects on tubulin and actin

In response to 1.0 ppm ATR, expression of the housekeeping genes actin and tubulin was reduced, as well as expression of GH and LH [Figure 1; see also Supplemental Material, Figure 2 (doi:10.1289/ehp.0900738)]. Therefore, to determine whether reduced gene expression might have resulted from cell toxicity or death, we used immunofluorescent staining to evaluate tubulin and actin microfilaments in pituitary cells treated with 1.0 ppm ATR. Although we observed some deterioration in filament structure (see Supplemental Material, Figure 3), the increase in PRL expression observed after ATR treatment (Figure 1) and the maintenance of relatively consistent levels of histone H3 expression (absolute levels are not shown) suggest that the cells were still functioning physiologically.

### GH protein levels in the GH3 cell line after blocking ACTH and PACAP receptors

**Figure 6:**
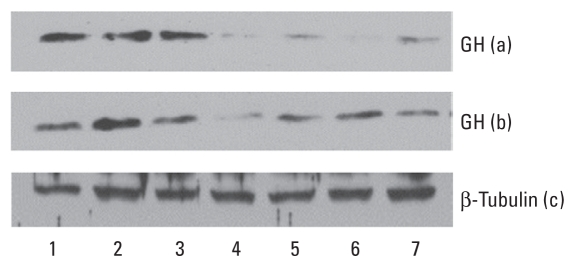
Levels of GH evaluated in rat GH3 pituitary cell culture 2 hr after ATR treatment. Lane 1, DMSO; lane 2, antibody (5 μL); lane 3, antibody (10 μL); lane 4, ATR (1 ppm); lane 5, ATR (1 ppb); lane 6, antibody (5 μL) and ATR (1 ppm); lane 7, antibody (10 μL) and ATR (1 ppm). The antibodies used were ACTHR (row a) and PACAP receptor (row b); tubulin (row c) was used as an internal loading control. Antibody alone served as a negative control.

ACTH protein has been reported to suppress GH secretion in blood serum (Izumi et al. 1985). To minimize the effect of ACTH on GH, we saturated rat GH3 pituitary cells with incremental amounts of ACTH receptor antibody before applying ATR. Consistent with our results using the AtT-20 cell line (Figure 3), we observed a remarkable reduction in GH levels after treatment with ATR at 1 ppm and 1 ppb (Figure 6). Furthermore, blocking the ACTH receptor with antibody did not interfere with the antagonistic effect of ATR on GH. We included the histone H3 antibody as a nonspecific antibody to either ACTH receptor or GHRHR, which served as a negative control [see Supplemental Material, Figure 4 (doi:10.1289/ehp.0900738)]. PACAP receptor has around 60% homology with GHRHR. Therefore, we used a similar approach to block possible binding of ATR to the PACAP receptor in GH3 cells using incremental amounts of PACAP receptor antibody. We observed a moderate alleviation of the antagonistic effect of ATR on GH protein levels (Figure 6).

## Discussion

In the present study we have identified a ligand-binding receptor, the GHRHR, which is responsive to the herbicide ATR. In a study on the distribution of ^14^C-ATR *in vivo* after lactational exposure in the Wistar rat, Stoker and Cooper (2007) showed that a small concentration of ATR was present in the anterior pituitary. This supports our finding that the pituitary gland is a target of ATR. The GHRH protein is produced in the hypothalamus and binds to the GHRHR in pituitary cells, resulting in the production of GH. GH regulates several metabolic processes in cells and differentiated tissues, including cell growth and proliferation; pituitary, prostate, and adrenal gland sizes; and the size of accessory reproductive organs (Cohen and Radovick 2002; Mogi et al. 2005; Stoker et al. 2002), which may explain the diverse effects noted in previous studies. GH also stimulates the activation of the gonadotropin-releasing hormone receptor, which leads to the secretion of gonadotropins and LH in pituitary cells (Childs et al. 1994b, 1999). We observed that *GH* and *LH* expression was reduced in primary cultured rat pituitary cells after exposure to ATR (Figure 1). Furthermore, our results suggest that *LH* expression may be reduced because of reduced *GH* expression resulting from ATR-mediated effects on the GHRHR. We also documented an increase in *PRL* expression in pituitary cells treated with ATR, which might be relevant to the mammary tumor formation in ATR-treated female rats reported by Wetzel et al. (1994). In another study, Giusi et al. (2006) found that ATR affected the dimorphic expression patterns of somatostatin subtype_2,3,5_ receptor mRNA. Somatostatin antagonizes the stimulatory actions of GHRH, which leads to repression of GH in mammals (Iranmanesh et al. 2004). Our findings suggest that increased somatostatin receptor expression may be secondary to an antagonistic effect of ATR on GHRHR. To our knowledge, this is the first evidence of a receptor in animals that binds the herbicide ATR. ATR may affect other pathways as well. We tested whether the effect of ATR on GH might also be mediated through the PACAP receptor. However, despite homology in protein domain sequences between the PACAP receptor and GHRHR, blocking ATR binding to the PACAP receptor had relatively moderate effects on GH expression in response to ATR, which suggests that ATR is not a primary ligand for the PACAP receptor.

## Conclusions

The results of the present study indicate that ATR targets the GHRHR of pituitary cells and that ATR-mediated inhibition of GH production in pituitary cells results from competition between ATR and GHRF for GHRHR binding. To our knowledge, this is the first time a receptor for ATR has been identified. GH is a major hormone of the endocrine system; thus, our results may help explain diverse effects of ATR reported by others. In addition, identification of GHRHR as a target of ATR may facilitate future studies of the effects of this herbicide on environmental and human health and inform efforts to develop guidelines for safe levels of ATR in water systems.

## References

[b1-ehp-118-1400] Arnold AP, Breedlove SM (1985). Organizational and activational effects of sex steroids on brain and behavior: a reanalysis. Horm Behav.

[b2-ehp-118-1400] Ashby J, Tinwell H, Stevens J, Pastoor T, Breckenridge CB (2002). The effects of atrazine on the sexual maturation of female rats. Regul Toxicol Pharmacol.

[b3-ehp-118-1400] Bloch B, Brazeau P, Ling N, Bohlen P, Esch F, Wehrenberg WB (1983). Immunohistochemical detection of growth hormone-releasing factor in brain. Nature.

[b4-ehp-118-1400] Carr JA, Gentles A, Smith EE, Goleman WL, Urquidi LJ, Thuett K (2003). Response of larval *Xenopus laevis* to atrazine: assessment of growth, metamorphosis, and gonadal and laryngeal morphology. Environ Toxicol Chem.

[b5-ehp-118-1400] Casale WL, Hart LP (1984). Influence of four herbicides on carpogenic germination and apothecium development of *Sclerotinia sclerotiorum*. Phytopathology.

[b6-ehp-118-1400] Childs GV, Unabia G, Miller BT (1994a). Cytochemical detection of GnRH binding sites on rat pituitary cells with LH, FSH, and GH antigens during diestrous upregulation. Endocrinology.

[b7-ehp-118-1400] Childs GV, Unabia G, Miller BT, Collins TJ (1999). Differential expression of gonadotropin and prolactin antigens by GHRH target cells from male and female rats. J Endocrinol.

[b8-ehp-118-1400] Childs GV, Unabia G, Rougeau D (1994b). Cells that express luteinizing hormone (LH) and follicle-stimulating hormone (FSH) β-subunit messenger ribonucleic acids during the estrous cycle: the major contributors contain LHβ, FSHβ, and/or growth hormone. Endocrinology.

[b9-ehp-118-1400] Cohen LE, Radovick S (2002). Molecular basis of combined pituitary hormone deficiencies. Endocr Rev.

[b10-ehp-118-1400] Connor K, Howell J, Chen I, Liu H, Berhane K, Sciarretta C (1996). Failure of chloro-*S*-triazine derived compounds to induce estrogenic receptor-mediated responses *in vivo* and *in vitro*. Fundam Appl Toxicol.

[b11-ehp-118-1400] Cooper RL, Stoker TE, Tyrey L, Goldman JM, McElroy WK (2000). Atrazine disrupts the hypothalamic control of pituitary-ovarian function. Toxicol Sci.

[b12-ehp-118-1400] Devos S, De Bosscher K, Staels B, Bauer E, Roels F, Berghe WV (2003). Inhibition of cytokine production by the herbicide atrazine search for nuclear receptor targets. Biochem Pharmacol.

[b13-ehp-118-1400] European Commission (2004). Decision Concerning the Non-inclusion of Atrazine in Annex I to the Council Directive 91/414/EEC and the Withdrawal of Authorisations for Plant Protection Products Containing This Active Substance. Official J Eur Union.

[b14-ehp-118-1400] Gammon DW, Aldous CN, Carr WC, Sanborn JR, Pfeifer KF (2005). A risk assessment of atrazine use in California: human health and ecological aspects. Pest Manag Sci.

[b15-ehp-118-1400] Giusi G, Facciolo RM, Canonaco M, Alleva E, Belloni V, Dessi-Fulgheri F (2006). The endocrine disruptor atrazine accounts for a dimorphic somatostatinergic neuronal expression pattern in mice. Toxicol Sci.

[b16-ehp-118-1400] Hayes E (1993). EPA’s chemical information database. EPA J.

[b17-ehp-118-1400] Hayes TB, Case P, Chui S, Chung D, Haeffele C, Haston C (2006). Pesticide mixtures, endocrine disruption, and amphibian declines: are we underestimating the impact?. Environ Health Perspect.

[b18-ehp-118-1400] Hayes TB, Collins A, Lee M, Mendoza M, Noriega N, Stuart A (2002a). Hermaphroditic, demasculinized frogs after exposure to the herbicide atrazine at low ecological relevant doses. Proc Natl Acad Sci USA.

[b19-ehp-118-1400] Hayes TB, Haston K, Tsui M, Hoang A, Haeffele C, Vonk A (2002b). Herbicides: feminization of male frogs in the wild. Nature.

[b20-ehp-118-1400] Hooghe RJ, Devos S, Hooghe-Peters EL (2000). Effects of selected herbicides on cytokine production *in vitro*. Life Sci.

[b21-ehp-118-1400] Huggett J, Dhehada K, Bustin S, Zumla A (2005). Real-time RT-PCR normalization; strategies and considerations. Genes Immun.

[b22-ehp-118-1400] Institute of Laboratory Animal Resources (1996). Guide for the Care and Use of Laboratory Animals.

[b23-ehp-118-1400] Iranmanesh A, Bowers CY, Veldhuis JD (2004). Activation of somatostatin-receptor subtype-2/-5 suppresses the mass, frequency, and irregularity of growth hormone (GH)-releasing peptide-2-stimulated GH secretion in men. J Clin Endocrin Metab.

[b24-ehp-118-1400] Izumi T, Imaizumi C, Ashida E, Ochiai T, Wang PJ, Fukuyama Y (1985). Suppressive action of ACTH on growth hormone secretion in patients with infantile spasms. Brain Dev.

[b25-ehp-118-1400] Kniewald J, Jakominic M, Tomljenovic A, Simic B, Romac P, Vranesic D (2000). Disorders of male rat reproductive tract under the influence of atrazine. J Appl Toxicol.

[b26-ehp-118-1400] Koskinen WC, Clay SA (1997). Factors affecting atrazine fate in north central US soils. Rev Environ Contam Toxicol.

[b27-ehp-118-1400] McMullin TS, Andersen ME, Nagahara A, Lund TD, Pak T, Handa RJ (2004). Evidence that atrazine and diaminochlorotirazine inhibit the estrogen/progesterone induced surge of luteinizing hormone in female Sprague-Dawley rats without changing estrogen reception action. Toxicol Sci.

[b28-ehp-118-1400] Mills PK (1998). Correlation analysis of pesticides use data and cancer incidence rates in California counties. Arch Environ Health.

[b29-ehp-118-1400] Missale C, Boroni F, Sigala S, Burrani A, Fabris M, Leon A (1996). Nerve growth factor in the pituitary: localization in mammotroph cells and cosecretion with prolactin by a dopamine-regulated mechanism. Proc Natl Acad Sci USA.

[b30-ehp-118-1400] Mogi C, God H, Mogi K, Takaki A, Yokoyama K, Tomida M (2005). Multistep differentiation of GH-producing cells from their immature cells. J Endocrinol.

[b31-ehp-118-1400] Montero M, Yon L, Kikuyama S, Dufour S, Vaudry H (2000). Molecular evolution of the growth hormone-releasing hormone/pituitary adenylate cyclase-activating polypeptide gene family. Functional implication in the regulation of growth hormone secretion. J Mol Endocrinol.

[b32-ehp-118-1400] National Center for Biotechnology Information (2010). GenBank Overview.

[b33-ehp-118-1400] O’Connor JC, Plowchalk DR, Van Pelt CS, Davis LG, Cook JC (2000). Role of prolactin in chloro-*S*-triazine rat mammary tumorigenesis. Drug Chem Toxicol.

[b34-ehp-118-1400] Ohyama T, Sato M, Ohye H, Murao K, Nimi M, Takahara J (1998). Effects of adrenalectomy and glucocorticoid receptor antagonist, RU38486, on pituitary growth hormone-releasing hormone receptor gene expression in rats. Peptides.

[b35-ehp-118-1400] Rekasi Z, Varga JL, Schally AV, Halmos G, Groot K, Czompoly T (2000). Antagonistic actions of analogs related to growth hormone-releasing hormone (GHRH) on receptors for GHRH and vasoactive intestinal peptide on rat pituitary and pineal cells *in vitro*. Proc Natl Acad Sci USA.

[b36-ehp-118-1400] Sagolio P, Trijasse S (1998). Behavioral responses to atrazine and diuron in goldfish. Arch Environ Contam Toxicol.

[b37-ehp-118-1400] Sagrillo CA, Elmanoff M (1998). Effects of prolactin on expression of the mRNAs encoding the immediate early genes *zif/268* (NGF1-A), *nur/77* (NGF1-B), c-*fos* and c-*jun* in the hypothalamus. Mol Brain Res.

[b38-ehp-118-1400] Shafer T, Ward TR, Meacham CA, Cooper RL (1999). Effects of the chlorotriazine herbicide, cyanazine, on GABA_A_ receptor in cortical tissue from rat brain. Toxicology.

[b39-ehp-118-1400] Spiess J, Rivier J, Vale W (1983). Characterization of rat hypothalamic growth hormone-releasing factor. Nature.

[b40-ehp-118-1400] Stoker TE, Cooper RL (2007). Distribution of ^14^C-atrazine following an acute lactational exposure in the Wistar rat. Reprod Toxicol.

[b41-ehp-118-1400] Stoker TE, Guidici DL, Laws SC, Cooper RL (2002). The effects of atrazine metabolites on puberty and thyroid function in the male Wistar rat. Toxicol Sci.

[b42-ehp-118-1400] Stoker TE, Robinette CL, Cooper RL (1999). Maternal exposure to atrazine during lactation suppresses sucking-induced prolactin release and results in prostatitis in the adult offspring. Toxicol Sci.

[b43-ehp-118-1400] Tamaki M, Sato M, Matsubara S, Wada Y, Takahara J (1996). Dexamethasone increases growth hormone (GH)-releasing hormone (GRH) receptor mRNA levels in cultured rat anterior pituitary cells. J Neuroendocrinol.

[b44-ehp-118-1400] Tennant MK, Hill DS, Eldridge JC, Wetzel LT, Breckenridge CB, Stevens JT (1994). Chloro-*S*-triazine antagonism of estrogen action: limited interaction with estrogen receptor binding. J Toxicol Environ Health.

[b45-ehp-118-1400] Thorner MO, Spiess J, Mary LV, Rogol AD, Kaiser DL, Webster JD (1983). Human pancreatic growth-hormone-releasing factor selectively stimulates growth-hormone secretion in man. Lancet.

[b46-ehp-118-1400] Trentacoste SV, Friedmann AS, Youker RT, Breckenridge CB, Zirkin BR (2001). Atrazine effects on testosterone levels and androgen-dependent reproductive organs in peripubertal male rats. J Androl.

[b47-ehp-118-1400] U.S. Environmental Protection Agency (2003). Notice of Revised Draft Ambient Water Quality Criteria Document for Atrazine and Request for Scientific Views. EPA Fact Sheet.

[b48-ehp-118-1400] Vandesompele J, De Preter K, Pattyn F, Poppe B, Van Roy N, De Paepe A (2002). Accurate normalization of real-time quantitative RT-PCR data by geometric averaging of multiple internal control genes. Genome Biol.

[b49-ehp-118-1400] Varga JL, Schally AV, Csernus VJ, Zarandi M, Halmos G, Groot K (1999). Synthesis and biological evaluation of antagonists of growth hormone-releasing hormone with high and protracted *in vivo* activities. Proc Natl Acad Sci USA.

[b50-ehp-118-1400] Velicelebi G, Patthi S, Kaiser ET (1986). Design and biological activity of analogs of growth hormone releasing factor with potential amphiphilic helical carboxyl termini. Proc Natl Acad Sci USA.

[b51-ehp-118-1400] Wetzel LT, Luempert LG, Breckendrige CB, Tisdel MO, Stevens JT, Thakur AK (1994). Chronic effects of atrazine on estrus and mammary tumor formation in female Sprague-Dawley and Fischer 344 rats. J Toxicol Environ Health.

[b52-ehp-118-1400] Withgott J (2002). Ubiquitous herbicide emasculates frogs. Science.

[b53-ehp-118-1400] Xu Y, Berelowitz M, Bruno J (1995). Dexamethasone regulates somatostatin receptor subtype messenger ribonucleic acid expression in rat pituitary GH4C1 cells. Endocrinology.

